# Exploring the use of wearable sensors for assessing risk factors during orthopedic surgeries: A protocol for data collection

**DOI:** 10.1016/j.mex.2024.102994

**Published:** 2024-10-09

**Authors:** Catarina Santos, Ana Teresa Gabriel, Cláudia Quaresma, Isabel L. Nunes

**Affiliations:** a UNIDEMI, Department of Mechanical and Industrial Engineering, NOVA School of Science and Technology, NOVA University Lisbon, Monte da Caparica, 2829-516 Caparica, Portugal; b Intelligent Systems Associate Laboratory, LASI, 4800-058 Guimarães, Portugal; c Laboratory for Instrumentation, Biomedical Engineering and Radiation Physics (LIBPhys-UNL), Physics Department, NOVA School of Science and Technology, NOVA University Lisbon, Monte da Caparica, 2829-516 Caparica, Portugal

**Keywords:** Lower limb, Musculoskeletal disorders, Electromyography, Inertial sensors, Physical exposure, Protocol for using wearable sensors to assess risk factors during orthopedic surgeries

## Abstract

During orthopedic surgeries, surgeons are generally exposed to prolonged periods of standing, awkward and sustained body postures, and forceful movements, which can increase the likelihood of developing work-related musculoskeletal disorders (WRMSD). Therefore, this study proposes a protocol to measure parameters related to physical risk factors contributing to lower limb WRMSD, during orthopedic surgery procedures. The protocol development was preceded by an initial phase of understanding and specifying the context of use, followed by pre-tests in laboratory environment. It integrates a motion capture system, using inertial measurement units (IMU) to collect posture data from hip, knee, and ankle, and electromyography system (EMG) to measure and record data from muscle activity of biceps femoris, rectus femoris, and gastrocnemius lateralis. Pre-tests provided insights for protocol optimization, estimating a 3-hour data collection session per surgery due to sensor battery limitations, streamlining the process by placing EMG sensors before IMU and refining thigh sensor placement strategies. The protocol presents an opportunity for a real-time and quantitative approach to monitor surgeon's exposure to risk factors contributing to lower limb WRMSD while performing surgical procedures. Two months after pre-tests, the protocol implementation began in a real work context. The study's final outcomes fall outside the paper's scope.

## Graphical abstract

**Figure:**
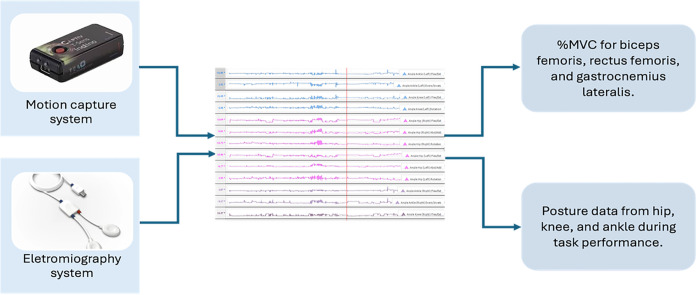

Specifications table

**Table:** 

Subject area:	Engineering
More specific subject area:	Ergonomics
Name of your protocol:	Protocol for using wearable sensors to assess risk factors during orthopedic surgeries
Reagents/tools:	CAPTIV from TEA EMG from PLUX
Experimental design:	The protocol development was preceded by an initial phase of understanding and specifying the context of use, which includes observations of surgical procedures, literature review, and interviews. After defining the protocol, pre-tests were conducted in laboratory environment, with 20 volunteers. The protocol integrates a motion capture system, using inertial measurement units (IMU) to collect posture data from hip, knee, and ankle, and surface electromyography system (EMG) to measure and record data from muscle activity of biceps femoris, rectus femoris, and gastrocnemius lateralis.
Trial registration:	N.A.
Ethics:	The protocol received approval from the Ethics committees of both NOVA FCT and CHULC. All participants provide their written informed consent prior to participation.
Value of the Protocol:	Provides real-time quantitative data to monitor surgeons' exposure to physical risk factors linked to lower limb WRMSD. Facilitates the continuous collection of procedural data and allows for the recording of lower limb postures without visual barriers from surgical instruments or equipment, thereby enhancing the accuracy of ergonomic risk assessments. By specifically targeting lower limb WRMSD, the protocol addresses a critical yet often overlooked aspect of occupational health concerns among orthopedic surgeons*.*

## Background

Work-related musculoskeletal disorders (WRMSD) constitute a significant occupational health concern, affecting the quality of life for approximately 60 % of the European workforce [1]. Particularly within the healthcare sector, WRMSD stand as a predominant cause of occupational disorders [2]. This notable prevalence underscores the critical need to address WRMSD and its associated risk factors.

Research on orthopedic surgical settings has revealed a high incidence of repetitive WRMSD and related symptoms [3]. Orthopedic surgery is generally characterized as a physically demanding endeavor, requiring prolonged maintenance of standing postures, adoption of awkward body postures, often recurring or persisting for extended durations, and enduring upright postures [4,5]. The cumulative impact of these work-related demands, compounded by insufficient time for the affected body regions to recover from fatigue, can precipitate the onset of WRMSD [6].

From an epidemiological perspective, orthopedic surgeons exhibit susceptibility to the development of WRMSD affecting the upper limbs, back, and lower limbs, as documented in recent studies [[3], [5]]. However, while WRMSD in the upper limbs and back have been well-documented, scientific literature regarding lower limb WRMSD remains limited and fragmented across various publications [7,8]. This fragmented understanding of the prevalence of lower limb WRMSD, its accompanying symptoms, and the underlying risk factors has restricted the understanding of the extent and implications of these disorders and their symptoms among orthopedic surgeons. Nonetheless, studies have highlighted that prolonged standing and maintaining sustained upright postures, especially during lengthy surgeries, are significant risk factors for the development of lower limb WRMSD [7,8]. Additionally, studies on other occupations requiring prolonged standing have consistently highlighted the importance of the duration of standing, working posture, muscle effort, and holding time conditions in the development of lower limb WRMSD [[9], [10], [11]].

Considering the critical role of lower limbs in maintaining body stability and balance during surgical operations, WRMSD affecting lower limbs could significantly compromise surgical precision and motor skills, potentially impacting the broader healthcare sector [[5], [12]]. A compelling illustration of this emerges from a study conducted by Yakkanti and colleagues, involving 1645 active orthopedic surgeons in the United States, which revealed that ankle or foot WRMSD carries the highest likelihood of triggering early retirement among orthopedic surgeons [13]. This underscores the importance of regular ergonomic assessments in orthopedic surgeries to identify risk factors and prevent lower limb WRMSD.

Traditionally, ergonomic risk assessments have heavily relied on self-reporting and observational techniques, lacking comprehensiveness and objectivity in assessing the exposure to risk factors [14]. Recent advances in wearable sensor technology allow a real-time and quantitative approach to monitoring surgeon's exposure to risk factors while performing surgical procedures [15]. While there is currently a shortage of studies exploring the integration of wearable sensors into ergonomic risk assessment methods applied to surgical procedures, promising studies have already integrated wearable sensors into ergonomic risk assessment methods applied to laparoscopic, urologic, otolaryngologic, and vascular surgeries [[15], [16], [17], [18], [19], [20], [21], [22], [23], [24], [25], [26]]. These studies primarily focus on collecting data specifically pertaining to upper limbs. The exception is the study conducted by Arrighi-Allisan and colleagues, which used IMU sensors to collect posture data from hip, knee, and ankle, assessing it with REBA method [16]. This highlights the gap in knowledge regarding collecting kinematic and EMG data to monitor exposure to risk factors related to lower limb WRMSD among orthopedic surgeons, hindering comprehensive ergonomic risk assessments and the development of preventive strategies for lower limb WRMSD within surgical procedures.

Hence, this study protocol aims to measure parameters related to physical risk factors contributing to the development of lower limb WRMSD, during orthopedic surgery procedures. The protocol encompasses the use of wearable sensors to provide a real-time quantitative approach to monitor exposure to awkward postures, repetitive movements, as well as to measure muscle activity parameters, regarding lower limbs.

## Description of protocol

### Methods

#### Study design

The protocol was developed in collaboration with the orthopedic team of Curry Cabral Hospital of Centro Hospitalar Universitário Lisboa Central (CHULC). It is based on observations conducted during orthopedic surgical procedures at the aforementioned hospital. These observations aimed to detail the tasks performed and analyze the main postures adopted by surgeons for each surgical task. Recognizing the inherent challenges of conducting research in a real operating theatre, particularly minimizing interference with the critical surgical workflow, the initial contact with the surgical teams was crucial. This collaboration enabled the development of a protocol prioritizing minimal disruption while ensuring the acquisition of data to assess the exposure to physical risk factors contributing to the development of lower limb WRMSD among orthopedic surgeons.

Additionally, a literature review was undertaken to identify the risk factors linked to the development of lower limb WRMSD among orthopedic surgeons and the ergonomic risk assessment methods integrating wearable sensors for data collection within surgical environments [27]. The main findings are presented in the introduction section.

**Fig. 1:**
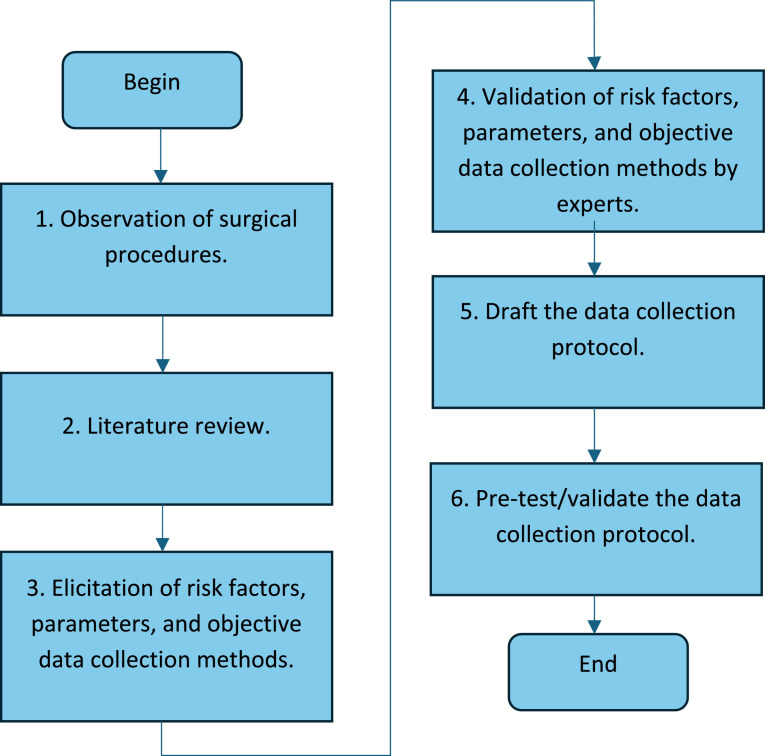
Methodology to define the protocol for data collection.

Subsequently, individual interviews were conducted with three occupational medical doctors. During these interviews, experts were asked to validate the identified risk factors, ascertain the parameters characterizing the risk factors and validate the objective data collection methods to measure them. The methodology employed is outlined in Fig. 1.

#### Equipment

The protocol integrates surface EMG sensors and a motion capture system with IMU sensors, providing a deeper and richer insight about physiological and kinematic parameters of the lower limbs. As previously mentioned, wearable sensors enable a real-time quantitative approach to monitor participant's exposure to risk factors during task performance.

**Fig. 2:**
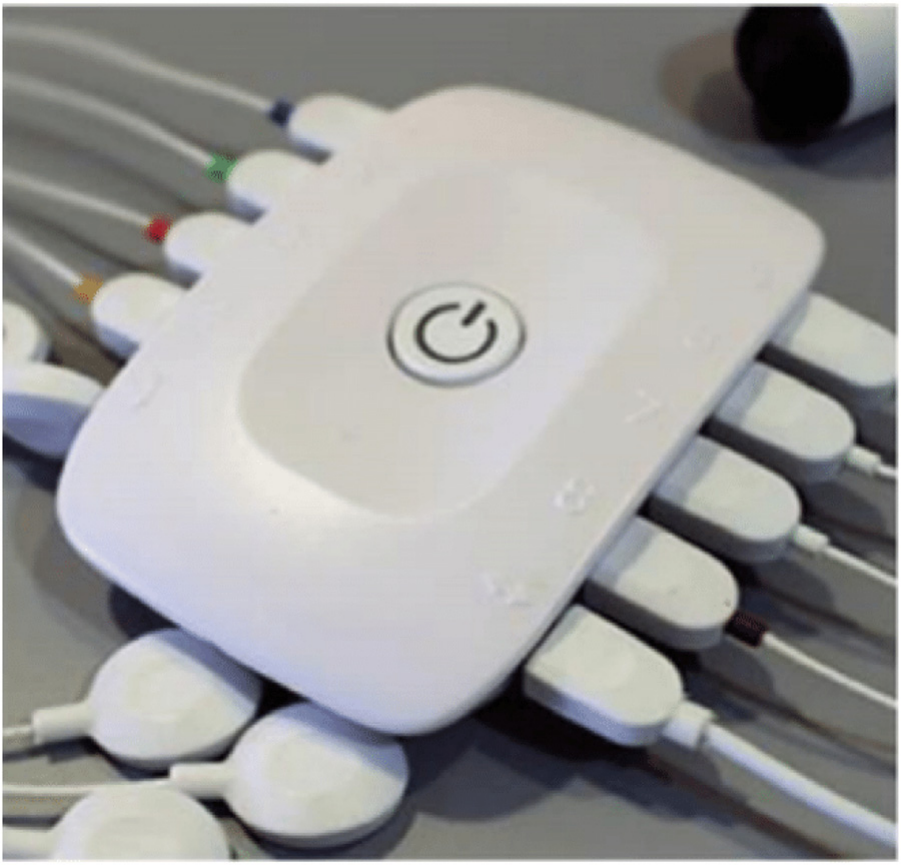
Biosignalplux system with the EMG sensors.

For EMG signal recording, a biosignalplux acquisition module is used, connected to seven surface EMG sensors from PLUX, as illustrated in Fig. 2. The multichannel EMG recording is performed on three muscles groups: the biceps femoris, rectus femoris, and gastrocnemius lateralis. Data is streamed over Bluetooth, sampled at frequency up to 1000 Hz, with a resolution of 16 bits. This bandwidth was chosen since EMG activity can go up to 500 Hz [28]. To connect the sensors to the participant, two Ag/AgCL with solid adhesive pre gelled electrodes are used per sensor. The data from biosignalplux is recorded using OpenSignals software.

**Fig. 3:**
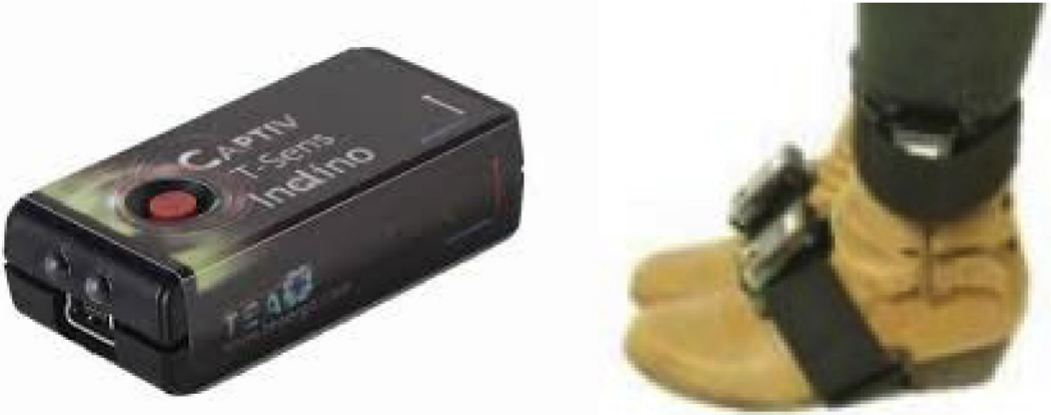
IMU wireless T-SENS motion sensors.

Conversely, kinematic data from hip, knees, and ankles is monitored using seven IMU wireless T-SENS motion sensors, such as the one depicted in Fig. 3, paired with 16-channel T-Rec wireless receiver with a range of 15 m, both from CAPTIV. Each IMU sensor, weighting 32 g and measuring 60 × 35 × 19 mm, integrates a tri-axial accelerometer, gyroscope, and magnetometer. The data is sampled at a frequency of 64 Hz, with a sensor's battery lifespan of up to 4 h. The collected data is recorded using CAPTIV software.

The selection of CAPTIV software was based on its recognized robust capabilities in human motion analysis. The software employs a quaternion-based method to calculate angles in the sagittal, frontal, and transversal planes during subject's recording. The study conducted by Peeters and colleagues compared the accuracy of the CAPTIV system to Vicon for capturing full-body joint angles during various motion tasks. Their findings demonstrated that IMU sensors from CAPTIV are reliable for the full body joint angles tracking in regular task as walking, but most of all in fast and complicated tasks like those encountered in practices in sports and rehabilitation [29]. For gait patterns, IMU sensors from CAPTIV demonstrated average root mean square errors (RMSE) of 1.9° for flexion/extension, 2.6° for abduction/adduction, and 3.7° for rotation angles. The corresponding average correlation coefficients were 0.87, 0.68, and 0.42 for each of these movements, respectively [29].

However, the study also highlighted differences between CAPTIV and Vicon in measuring shoulder, wrist, and back angles, making direct comparisons challenging [29]. For instance, the shoulder angles measured by the IMU sensors from CAPTIV are similar to Vicon's, differing only by ±2π. Additionally, CAPTIV measures wrist flexion/extension relative to the forearm, while Vicon measures it relative to the body's sagittal plane. CAPTIV also simplifies back flexion by treating the thoracic and cervical spine as a single unit, whereas Vicon distinguishes between the two. These differences make RMSE and correlation coefficients less reliable when comparing shoulder, wrist, and back angles. Despite these issues, CAPTIV's joint angle measurements are generally accurate within 5°, except for elbow flexion/extension during wrist rotation, where an RMSE of 6.4° was reported [29].

The study also concluded that while CAPTIV's accuracy declines slightly during fast or complex movements (with RMSE ≤ 8.0°), the results remain within acceptable limits, making it suitable for a wide range of tasks [29].

In another study, Steinebach and colleagues evaluated CAPTIV's accuracy in measuring upper limb joint angles [30]. By comparing it to a goniometer for static postures and an angle scale for dynamic movements, they found that CAPTIV achieved high accuracy, with correlation coefficients above 0.93 and mean absolute errors under 5° for most movements, except for elbow flexion [30].

#### Protocol

The initial step of the data acquisition protocol is positioning the electrodes in three muscles groups, in both lower limbs: the biceps femoris, rectus femoris, and gastrocnemius lateralis. The muscle groups were selected based on the observations of surgeon's body postures during surgical procedures. This selection was made in close collaboration with the orthopedic team at Curry Cabral Hospital.

The biceps femoris muscle is responsible for flexion and lateral rotation of the knee joint, as well as hip extension and lateral rotation (via the long head). The rectus femoris muscle is responsible for knee extension and hip flexion. The gastrocnemius lateralis muscle is responsible for ankle flexion and assists with knee flexion.

These movements are important for maintaining postural control and allowing dynamic adjustments during surgery. In addition to their functional importance, practical considerations were taken into account to minimize disruption to the surgical process. Monitoring these three muscle groups per lower limb allowed for meaningful data collection while avoiding sensor's overload, thus reducing discomfort and maintaining the surgeon's mobility.

To capture muscle activity data, two electrodes are placed on each muscle, two centimeters apart on the muscle belly and aligned with the muscle fibers according to established guidelines provided by SENIAM [31]. For biceps femoris, the electrodes are placed at the midpoint of the line between the ischial tuberosity and the lateral epicondyle of the tibia. Regarding the rectus femoris, electrodes are positioned at the midpoint of the line between the anterosuperior iliac spine and the patella. For the gastrocnemius lateralis, the electrodes are placed one-third of the distance between the head of the fibula and the heel. Additionally, a reference electrode is placed at the external malleolus.

Following electrode placement, baseline muscle activity is recorded. Therefore, the maximum voluntary contractions (MVC) for each muscle needs to be determined, which requires performing three different tasks [32].

**Fig. 4:**
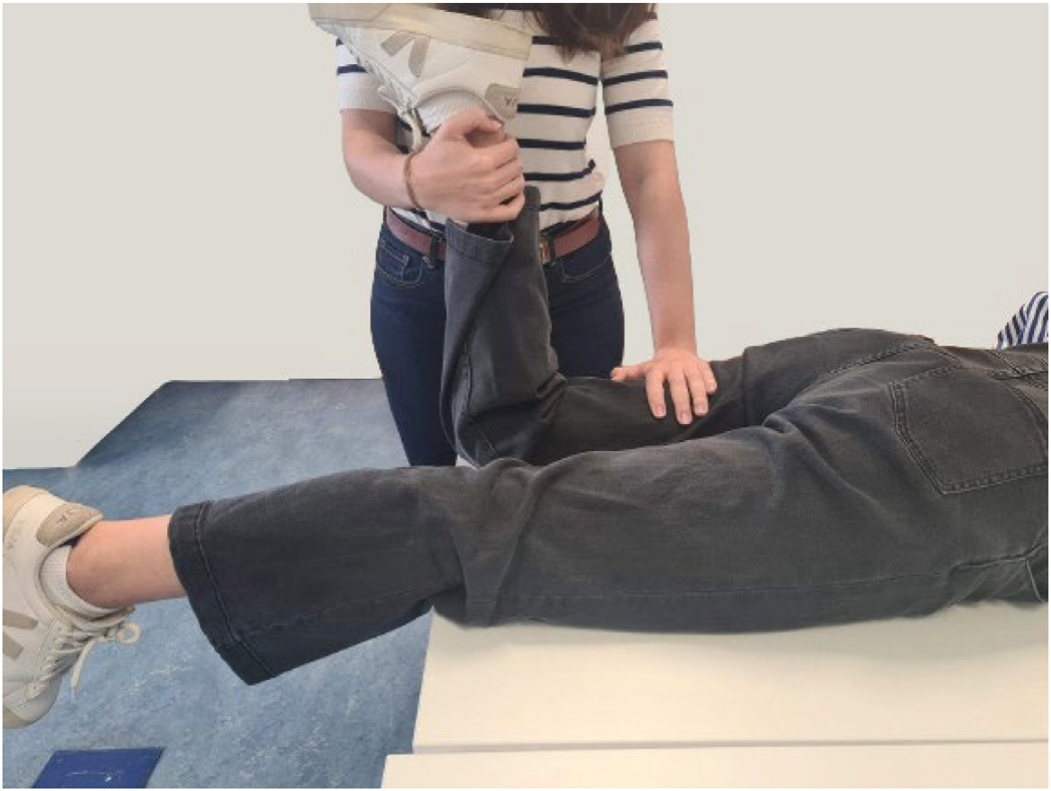
MVC baseline assessment for biceps femoris.

For the biceps femoris, the participants assumed a prone posture with the knee flexed at approximately 45° and the leg externally rotated, while the researcher applied pressure to the ankle region to counteract knee flexion, as shown in Fig. 4.

**Fig. 5:**
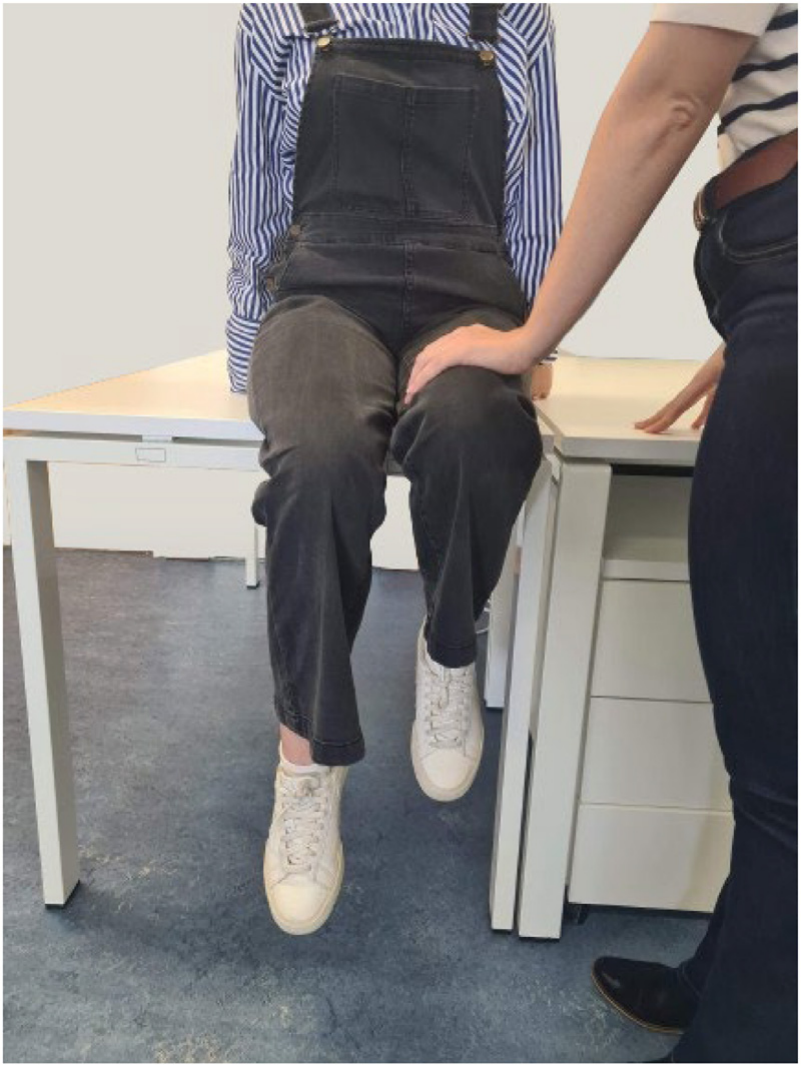
MVC baseline assessment for rectus femoris.

For the rectus femoris, the participants sit with thighs fully supported on the table, utilizing upper limbs for trunk stability by gripping the table's edge or placing hands on either side. The task involves hip flexion while maintaining neutral rotation, with the researcher providing resistance to counteract the movement, as shown in Fig. 5.

**Fig. 6:**
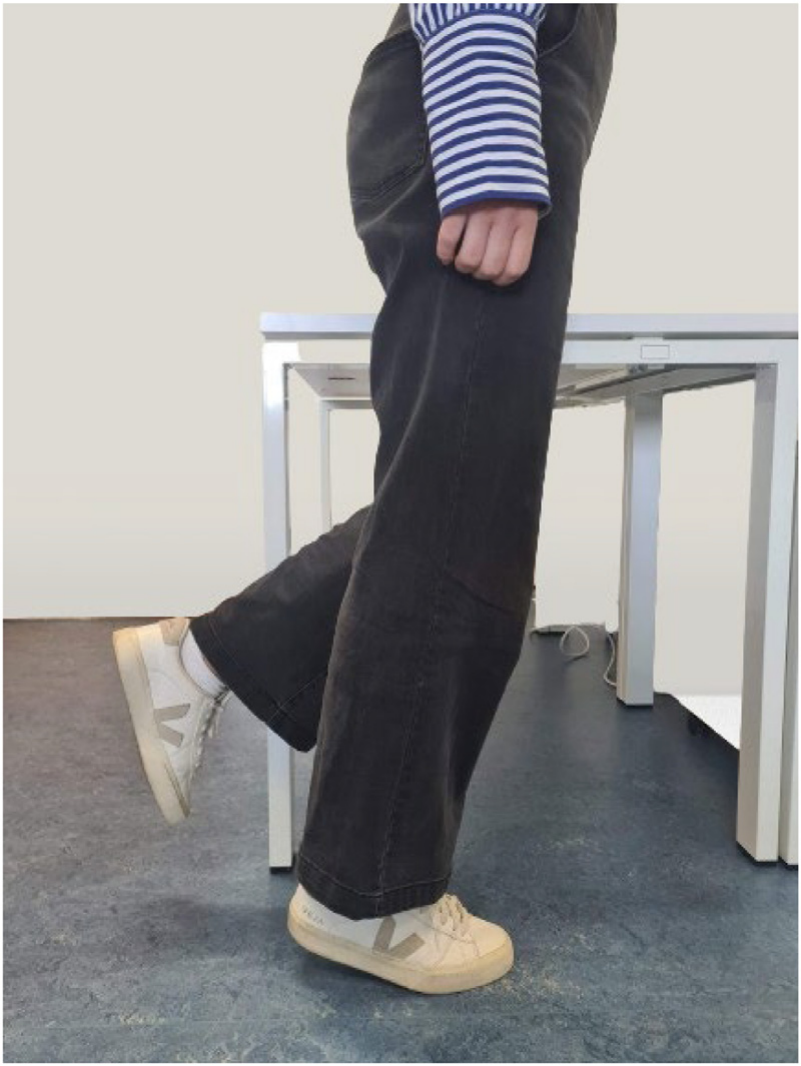
MVC baseline assessment for gastrocnemius lateralis.

For the gastrocnemius lateralis, the participants stand with weight on the limb to be tested, possibly resting fingers on a table for support. The participants are instructed to perform plantar flexion on the tested limb to achieve maximum amplitude, as shown in Fig. 6. The task is repeated three times.

After determining the MVC for each muscle, participants are asked to place seven IMU wireless T-SENS motion sensors to measure hip, knees, and ankles kinematic data, following manufacturer's guidelines and using specific adjustable straps.

**Fig. 7:**
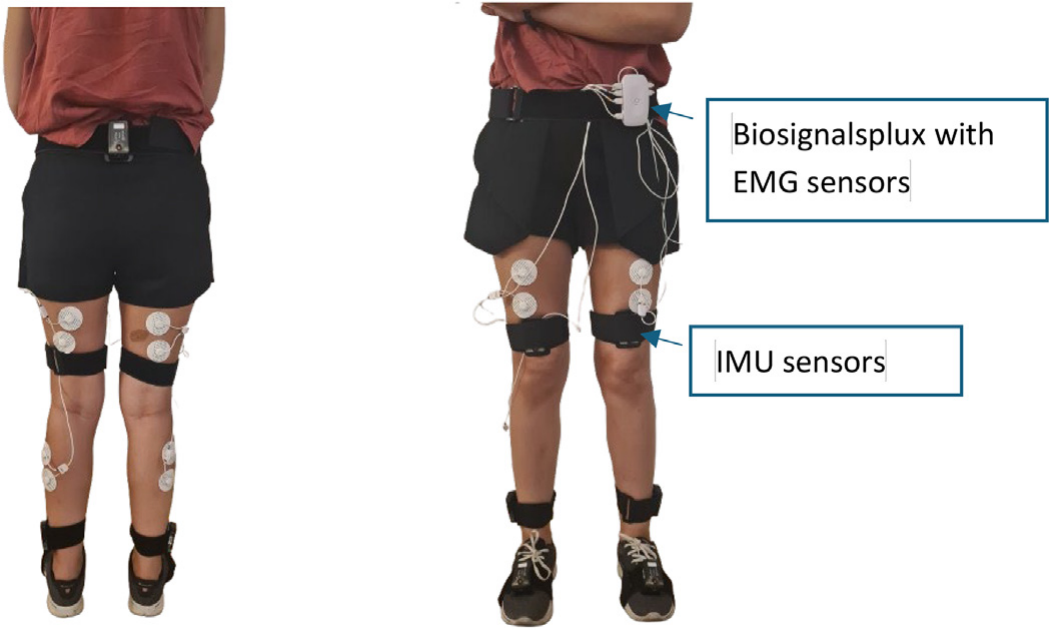
Illustration of the placement of the IMU sensors and EMG sensors.

Three IMU sensors are required for measuring knee and ankle's posture. The sensor for the thigh is placed on the upper leg facing forward above the knee, the sensor for the lower leg is placed facing outward above the ankle, and the sensor for the foot is placed on top of the foot using the supplied strap or in the shoelaces. Additionally, one IMU sensor is positioned behind the participant's back, centered on the sacrum, essential for recording hip's posture and facilitating accurate 3D visualization during lower limb tracking. Fig. 7 illustrates the positioning of both IMU and surface EMG sensors.

Subsequently, IMU sensors are calibrated using two standing postures, each held for 6 s. The first posture involves the upper limbs alongside the body, torso in neutral posture, head in neutral posture looking towards the horizon, feet parallel hip-width apart. The second posture entails extended upper limbs perpendicular to the torso, palms perpendicular to the ground, and bent legs.

Following IMU sensor calibration, which is conducted before the scrubbing process, data recording begins when the surgeon positions the patient for the start of the surgery.

To validate the protocol, a pre-test was conducted in a laboratory setting, where participants performed seven tasks based on observations conducted at Curry Cabral Hospital. These tasks were performed in a standing posture with intervals of 1 min rest and are as follows:

• Supporting a weight of 2 kg with both hands and slight trunk flexion, up to 20°, for 5 min. This task simulates the action of holding the patient's lower limb during surgery.
• Simulating sewing activity on the table for 5 min. This task simulates the surgical closing procedure, specifically the wound suturing performed by the surgeon.
• Simulating hammering activity on the table while rotating the trunk and keeping the feet staggered. The left foot should be placed forward and supported on the ground, and the right foot in plantar flexion with flexion of the right knee up to 35°, and slight extension of the hip up to 15°. The activity is performed 15 times. This task simulates the placement of the prosthesis in patients.
• Simulating hammering activity on the table while keeping the body close to the table with the lower limbs close to each other, and the feet parallel and anteriorly facing. The activity is performed 15 times and simulates the placement of the prosthesis.
• Trunk flexion over the table with hands supported on the table, with shoulder flexion and arms fully extended. The lower limbs should be in a neutral posture with the feet anteriorly facing and with plantar flexion of the left foot for 5 min. This task simulates the procedures observed during the placement of a prosthesis on the patient's lower limb.
• Trunk flexion over the table with hands supported on the table, and arms fully extended. The lower limbs should be in a neutral posture with the feet anteriorly facing and plantar flexion of both feet for 5 min. This task simulates the tasks observed during the placement of the prosthesis on the patient's lower limb.
• Lateral displacement with manual handling of loads from one table to another. The tables are arranged perpendicular, with the participant initially facing the table where a box is placed. The participant should carry it close to the body with elbows flexed at 90° in the sagittal plane and hands on each side of the box. The participant should move laterally facing the table and when approaching the second table, should rotate the trunk and squat to place the object on the table, which is positioned on the left side. The activity is performed 15 times. This task simulates the surgeon's interaction with surgical instruments and equipment during the procedure.

The protocol received approval from the Ethics committees of both NOVA FCT and CHULC.

#### Pre-test participants

The pre-test was conducted in the ergonomics laboratory of the Department of Mechanical and Industrial Engineering at NOVA FCT. The protocol was tested with 20 volunteers aged between 18 and 65 years, without associated pathology. 60 % of the participants were female, with a mean age of 30.1 years and a standard deviation of 7.9 years. All participants provided their written informed consent prior to participation.

### Data recording and analysis

As previously mentioned, the EMG and the kinematic data are recorded simultaneously using two different systems, each with its software installed on the same computer.

Prior to data collection IMU sensors undergo initialization. This procedure involves sensor calibration through calculation of cumulative orientation differences, requiring a magnetically neutral environment (i.e., free of metal or magnets) to ensure accuracy. Sensors can only be used for measurement after a positive test.

During the data recording, a trigger is inserted in the CAPTIV system to demarcate the onset and conclusion of each task. The seven tasks outlined in subsection 2.3 are marked during the pre-test phase. For surgical procedures, the tasks are determined in collaboration with surgeons prior to data acquisition, ensuring they reflect the surgical steps involved.

In addition to the data recorded by OpenSignals and CAPTIV software, and to ensure comprehensive documentation of all orthopedic surgeries, the researcher records the surgery type and patient's body posture on the operating table during surgery.

Regarding the data analysis, the kinematic data undergoes pre-processing in the CAPTIV software to convert rotation quaternions into joint angles in degrees. Then, the csv files containing EMG data from MVC procedure and tasks are exported from the OpenSignals and imported into CAPTIV. This process ensures the synchronization of the EMG and kinematic data.

Further processing of the EMG signal is conducted within CAPTIV. First, the EMG signal is averaged out and the signal envelope is extracted using the root mean square (RMS) algorithm, with a window of 100 samples. Each muscle RMS signal is then normalized using the maximum value of the respective MVC to obtain the %MVC for each muscle.

**Fig. 8:**
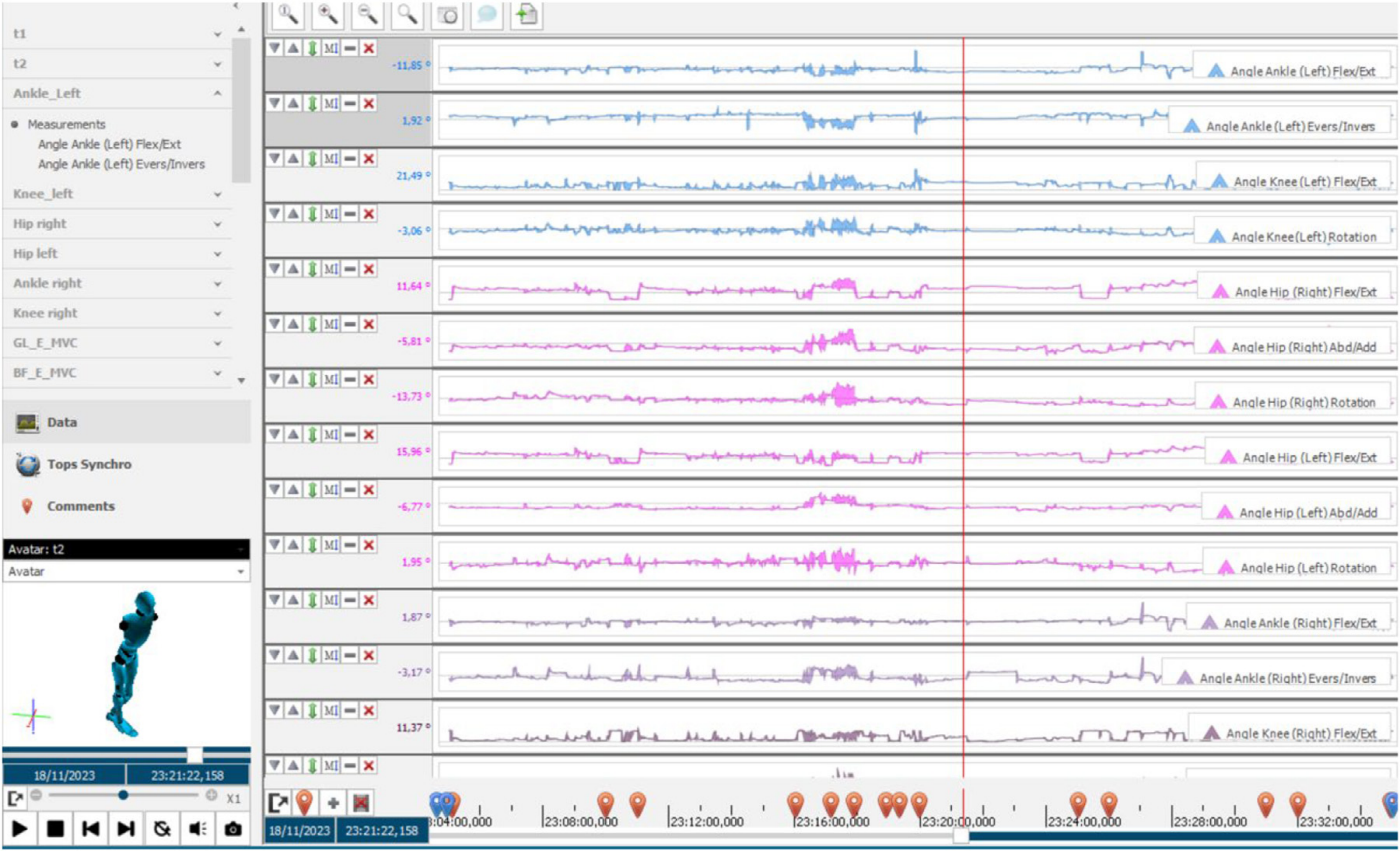
The captured data with CAPTIV sensor system, with synchronization of data and avatar visualization.

The main outcomes of this protocol will include %MVC for biceps femoris, rectus femoris, and gastrocnemius lateralis, alongside joint angle measurements during task performance. The angles are associated with hip flexion/extension, hip internal/external rotation, hip abduction/adduction, knee flexion, knee internal/external rotation, ankle dorsi/plantar flexion, ankle inversion/eversion during the task's performance. These outputs can be exported from CAPTIV software and saved as csv file. In addition, CAPTIV software allows the visualization of a 3D avatar representing the collected kinematic data. Fig. 8 illustrates the captured data with CAPTIV system.

## Protocol validation

### Results

This section outlines the findings from the pre-test phase, aimed at validating the protocol. The results provided insights into the duration of data collection, estimated at approximately 3 h per surgery due to sensor's battery limitations. After consecutive pre-tests, it was observed that the battery consistently lasted for the intended duration.

Additionally, the pre-tests highlighted the importance of placing the surface EMG sensors before IMU sensors, as the latter requires an initialization that may consume some time due to magnetic interferences. To streamline the process, the researcher optimized efficiency by initializing the IMU sensors while participants were placing the surface EMG sensors.

Furthermore, the pre-test helped to optimize thigh sensor placement. TEA-ergo recommendation was to position the sensor outwards on the upper leg for standing postures and facing forward for sitting postures. However, this standing posture placement hampered the IMU sensors calibration process. Consequently, the thigh sensor placement remained in the forward-facing position despite surgeons working in standing postures.

Ensuring accurate positioning of IMU sensors and firm attachment via the straps emerged as paramount, as deviations could compromise data acquisition accuracy. This was evident through incorrect avatar representation in IMU sensor calibration.

In some pre-test sessions, connectivity issues with the EMG sensors arose, interrupting signal acquisition. In such cases, the recorded signal was saved, and another recording session was immediately initiated. Post-data acquisition, the EMG files were concatenated chronologically to minimize data gaps.

Lastly, participants reported no discomfort while using the sensors, indicating overall feasibility and acceptability of the protocol.

### Discussion

As far as authors are aware, this is the first protocol integrating surface EMG sensors and a motion capture system with IMU sensors to monitor surgeons’ lower limbs during orthopedic surgeries. This approach employs real-time, quantitative data to monitor surgeons’ exposure to physical risk factors linked to the development of lower limb WRMS, supporting the ergonomic risk assessment of surgical tasks.

The integration of wearable sensors offers significant advantages over self-reporting and observational data collection methods, allowing for the monitoring of both physiological and kinematic parameters. By capturing data during surgery, this protocol enables an objective quantification of exposure to awkward postures, repetitive movements, as well as the measurement of muscle activity parameters. Moreover, the use of wearable sensors enables the collection of continuous procedural data, rather than snapshots, facilitating a more accurate basis for ergonomic risk assessments [15,21]. Notably, wearable sensors enable the recording of lower limb postures without visual barriers posed by surgical instruments, equipment, and other surgical team members [18]. Furthermore, focusing the protocol solely on the lower limbs ensures that sensors do not interfere with sterile scrubbing procedures, thus maintaining the integrity of the surgical environment [21].

Despite its potential benefits, implementing the protocol in real surgical settings presents several challenges. Foremost among these is the need to reduce interference with surgical procedures during data collection. Given the precision and concentration required for surgical tasks, careful planning and coordination with surgical teams are imperative to ensure smooth data collection. Thus, the development of the protocol involved a multi-step approach, including observations of surgical procedures, literature review, expert interviews, and pre-testing. Collaboration with surgical teams was essential to reduce disruption to workflow during data collection. Furthermore, individual interviews with occupational medicine doctors helped validate the identified risk factors, ascertain the parameters characterizing the risk factors and validate data collection methods. Also, testing the protocol in a controlled setting allowed for refinement before real-world application. Key considerations included the duration of data collection, sensor placement sequence, and calibration procedures.

Additionally, the protocol's reliance on wearable sensors entails addressing technical challenges. These include limitations such as sensor's battery life, electromagnetic interference, Bluetooth connectivity issues, the need to repeat IMU calibration each time the kinematic model had a slight orientation and postural drift, as well as potential errors stemming from skin movement or sensor drop [[15], [16],^19^]. To mitigate these challenges, measures such as securely fastening straps and using adhesive tape to affix IMU sensors to shoes can help reduce errors related to sensor placement. Additionally, it's worth noting that participants' awareness of the sensors may influence their posture, as observed in a study conducted by Arrighi-Allisan and colleagues, which evaluates surgeons performing functional endoscopic sinus surgery [16].

Nevertheless, the outcomes of this study protocol may have significant implications for occupational health and safety practices within the healthcare sector. By collecting data on parameters characterizing risk factors contributing to lower limb WRMSD among orthopedic surgeons, the protocol outcomes empower surgeons to make informed decisions during surgery, effectively mitigating their exposure and reducing the likelihood of lower limb WRMSD occurrence.

This protocol is currently being implemented at Curry Cabral Hospital during orthopedic surgeries. The participants belong to the orthopedic service team and perform surgeries in standing posture. Additionally, surgeries exceeding 3 h are excluded due to sensor battery limitations. The collected data will be used to assess the physical risk factors using a fuzzy decision support model. The model is an adaptation of ERGO-X to determine the possibility of orthopedic surgeons developing lower limb WRMSD for each limb, as consequence of performing surgical tasks [33,34].

Each risk factor is assessed based on attributes suitable to determine the severity of the risk factor. For instance, when considering the joint posture risk factor evaluation, the attributes are the specific joint possible movements (e.g., abduction/adduction, flexion/extension, internal/external rotation for hip joint; flexion, internal/external rotation for knee joint; or plantar/dorsiflexion, inversion/eversion for ankle joint), which are based on the data obtained from inertial sensors. Muscle effort is assessed by estimating the static, maximum, and minimum contraction levels, derived from the amplitude probability distribution function, which is calculated using EMG data from the biceps femoris, rectus femoris, and gastrocnemius lateralis [35]. For the static standing duration, the attribute is the time spent in continuous static standing work, which can be obtained from CAPTIV software.

The objective data used to evaluate the attributes are converted, using a continuous fuzzy set, in attribute's inadequacy degrees and then aggregated, using union fuzzy operators, into the respective physical risk factor's inadequacy degrees. The definition of the membership functions that characterize the fuzzy sets is based on the available literature and occupational physician's opinions. The results of inadequacy degrees are presented quantitatively, as membership degrees to an inadequacy fuzzy set, defined in the range [0, 1].

**Fig. 9:**
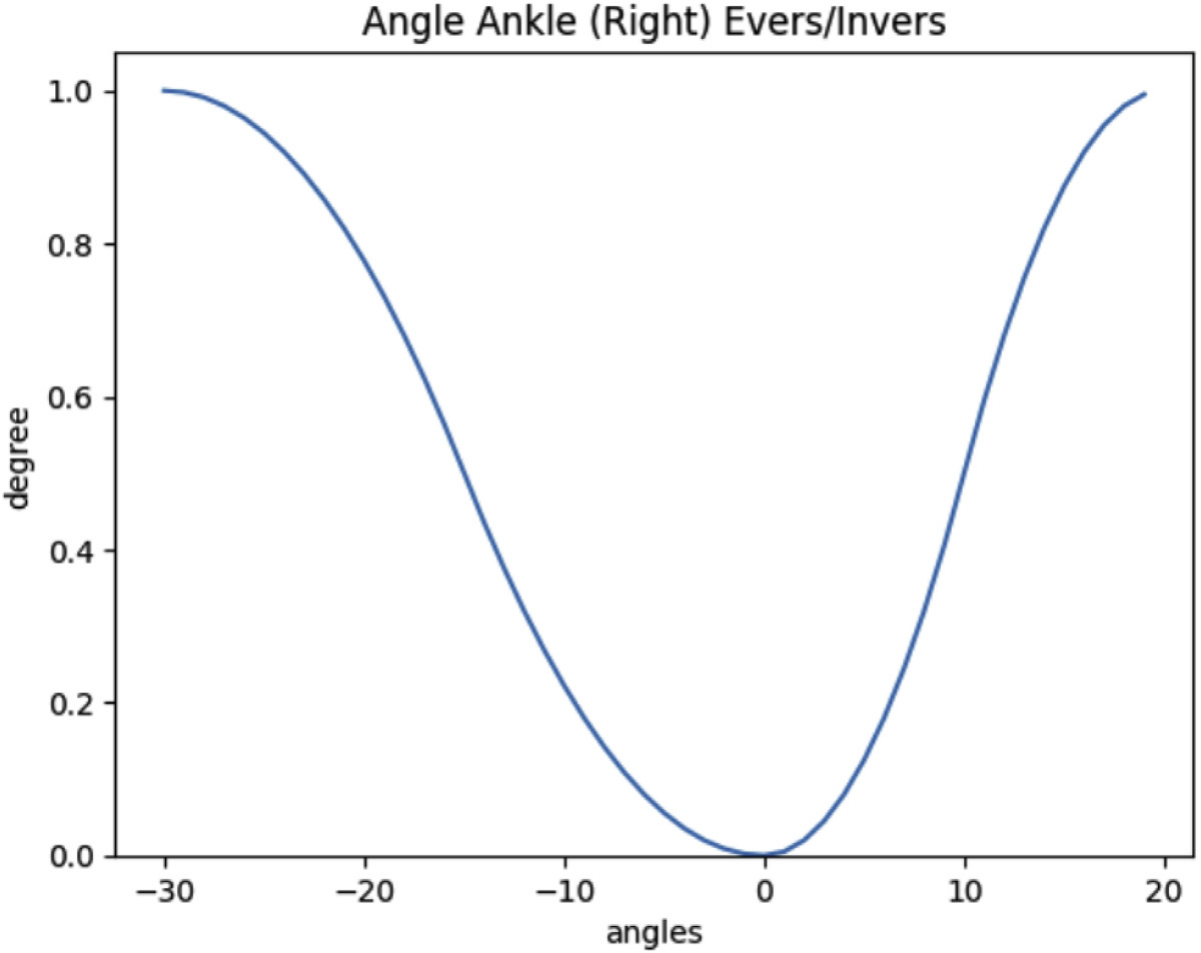
Example of a membership function of the ankle eversion/inversion angle, considering *t* = 0.

For instance, the membership function for a joint's movement is defined based on the range of motion. In the case of ankle eversion/inversion, which is represented in Fig. 9, inversion angles below −30° and eversion angles exceeding 20° are considered extreme postures, resulting in an entirely inappropriate posture. Angular values falling between these two thresholds represent intermediate inadequacy degrees, with the neutral instantaneous posture at 0° considered entirely suitable, resulting in an inadequacy degree of zero. These membership functions are further adjusted according to the surgeon's exposure time.

To ensure consistency in evaluating joint movements along a common axis, such as eversion and inversion, flexion and extension, abduction and adduction, external and internal rotation, it was established that positive angles would be attributed to flexion, abduction, eversion, and external rotation while negative angles would correspond to extension, adduction, inversion, and internal rotation.

For muscle effort, the continuous membership function is an S-type curve, where the inadequacy degree is zero at 0 %MVC and reaches one at 100 %MVC. Similarly, for static standing duration, the continuous membership function is S-type, with an inadequacy degree of zero at zero hours, increasing to one at one hour, which is the maximum recommended duration for continuous standing [36].

The possibility of developing lower limb WRMSD arises from the aggregation of the physical, individual, and psychosocial risk factor's inadequacy degrees, considering the weighting factors of the contribution of each risk factor for the development of lower limb WRMSD. The aggregation uses union fuzzy operators, enabling the simulation of synergy effects. Lastly, the fuzzy result goes through a defuzzification process, which uses linguistic variables to generate conclusions about the possibility of developing a lower limb WRMSD of each limb, for each task. For the relevant tasks, the computed risk factors and attribute's inadequacy degrees are analyzed to identify the physical risk factors and respective attributes contributing the most to the overall result.

In the future, the assessment results of the fuzzy decision support model are expected to contribute to the occupational safety and well-being of orthopedic surgeons.

### Conclusion

The development of the described protocol represents a significant advancement in addressing the occupational health concerns regarding lower limb WRMSD among orthopedic surgeons.

The proposed protocol allows to collect objective data for assessing the physical risk factors during orthopedic surgeries. The protocol includes surface EMG sensors and a motion capture system, integrating IMU sensors, that can provide real-time monitorization of physiological and kinematic parameters. Moreover, the protocol ensures no interference with sterile scrubbing procedures, thereby preserving the surgical environment's integrity and ensuring compatibility with the demanding nature of surgical tasks, which necessitate precision and concentration.

Although the protocol may pose some challenges associated with wearable sensor technology, its potential benefits are profound. In essence, the use of this protocol represents a promising step forward in providing effective means to support mitigating WRMSD risk factors for surgeons and fostering a safer, healthier work environment within the surgical field.

## Limitations

Not applicable.

## CRediT authorship contribution statement

**Catarina Santos:** Conceptualization, Methodology, Formal analysis, Investigation, Writing – original draft. **Ana Teresa Gabriel:** Conceptualization, Writing – review & editing, Supervision. **Cláudia Quaresma:** Conceptualization, Writing – review & editing, Supervision. **Isabel L. Nunes:** Conceptualization, Writing – review & editing, Supervision.

## Declaration of competing interest

The authors declare that they have no known competing financial interests or personal relationships that could have appeared to influence the work reported in this paper.

## Data Availability

Data will be made available on request.
